# High prevalence and risk of malaria among asymptomatic individuals from villages with high prevalence of artemisinin partial resistance in Kyerwa district of Kagera region, north-western Tanzania

**DOI:** 10.1186/s12936-024-05019-5

**Published:** 2024-06-26

**Authors:** Salehe S. Mandai, Filbert Francis, Daniel P. Challe, Misago D. Seth, Rashid A. Madebe, Daniel A. Petro, Rule Budodo, Angelina J. Kisambale, Gervas A. Chacha, Ramadhan Moshi, Ruth B. Mbwambo, Dativa Pereus, Catherine Bakari, Sijenunu Aaron, Daniel Mbwambo, Abdallah Lusasi, Stella Kajange, Samuel Lazaro, Ntuli Kapologwe, Celine I. Mandara, Deus S. Ishengoma

**Affiliations:** 1 National Institute for Medical Research, Dar es Salaam, Tanzania; 2 National Institute for Medical Research, Tanga Research Centre, Tanga, Tanzania; 3 University of Dar es Salaam, Dar es Salaam, Tanzania; 4 National Malaria Control Programme, Dodoma, Tanzania; 5 President’s Office, Regional Administration and Local Government, Dodoma, Tanzania; 6 Directorate of Preventive Services, Ministry of Health, Dodoma, Tanzania; 7 Faculty of Pharmaceutical Sciences, Monash University, Melbourne, Australia; 8 Harvard T.H Chan School of Public Health, Harvard University, Boston, MA USA; 9 Department of Biochemistry, Kampala International University in Tanzania, Dar es Salaam, Tanzania

**Keywords:** Malaria, Asymptomatic infections, *Plasmodiumfalciparum*, Predictors/risk factors of malaria infections, Artemisinin partial resistance, Kelch 13, Tanzania

## Abstract

**Background:**

Although Tanzania adopted and has been implementing effective interventions to control and eventually eliminate malaria, the disease is still a leading public health problem, and the country experiences heterogeneous transmission. Recent studies reported the emergence of parasites with artemisinin partial resistance (ART-R) in Kagera region with high prevalence (> 10.0%) in two districts of Karagwe and Kyerwa. This study assessed the prevalence and predictors/risk of malaria infections among asymptomatic individuals living in a hyperendemic area where ART-R has emerged in Kyerwa District of Kagera region, north-western Tanzania.

**Methods:**

This was a community-based cross-sectional survey which was conducted in July and August 2023 and involved individuals aged ≥ 6 months from five villages in Kyerwa district. Demographic, anthropometric, clinical, parasitological, type of house inhabited and socio-economic status (SES) data were collected using electronic capture tools run on Open Data Kit (ODK) software. Predictors/risks of malaria infections were determined by univariate and multivariate logistic regression, and the results were presented as crude (cORs) and adjusted odds ratios (aORs), with 95% confidence intervals (CIs).

**Results:**

Overall, 4454 individuals were tested using rapid diagnostic tests (RDTs), and 1979 (44.4%) had positive results. The prevalence of malaria infections ranged from 14.4% to 68.5% and varied significantly among the villages (p < 0.001). The prevalence and odds of infections were significantly higher in males (aOR = 1.28, 95% CI 1.08 –1.51, p = 0.003), school children (aged 5–≤10 years (aOR = 3.88, 95% CI 3.07–4.91, p < 0.001) and 10–≤15 years (aOR = 4.06, 95% CI 3.22–5.13, p < 0.001)) and among individuals who were not using bed nets (aOR = 1.22, 95% CI 1.03–1.46, p = 0.024). The odds of malaria infections were also higher in individuals with lower SES (aOR = 1.42, 95% CI 1.17–1.72, p < 0.001), and living in houses without windows (aOR = 2.08, 95% CI 1.46–2.96, p < 0.001), partially open (aOR = 1.33, 95% CI 1.11–1.58, p = 0.002) or fully open windows (aOR = 1.30, 95%CI 1.05–1.61, p = 0.015).

**Conclusion:**

The five villages had a high prevalence of malaria infections and heterogeneity at micro-geographic levels. Groups with higher odds of malaria infections included school children, males, and individuals with low SES, living in poorly constructed houses or non-bed net users. These are important baseline data from an area with high prevalence of parasites with ART-R and will be useful in planning interventions for these groups, and in future studies to monitor the trends and potential spread of such parasites, and in designing a response to ART-R.

**Supplementary Information:**

The online version contains supplementary material available at 10.1186/s12936-024-05019-5.

## Background

In the past two decades, there have been enhanced malaria control efforts that have resulted in a significant decline in the disease burden globally, but progress has stalled since 2015 [1]. According to the World Health Organization (WHO), there were an estimated 249 million malaria cases and 608,000 deaths in 2022, with continued limited progress since 2015 [2]. The WHO African Region (WHO-Afro) had the highest number of malaria cases and deaths, with an estimated 233 million cases (93.6%) and 580,000 deaths (95.4%) in 2022 [2]. Although the global scale-up of effective malaria interventions has saved millions of lives globally and cut malaria mortality by 36.0% from 2010 to 2020, the resulting optimism leading to hopes and plans to eliminate and ultimately eradicate malaria is facing imminent threats. The biggest contemporary challenges to malaria elimination include insecticide resistance by malaria vectors [3, 4], emergence and spread of drug resistance [5, 6], the emergence of parasites that cannot be detected by rapid diagnostic tests (RDTs) due to deletions of the histidine-rich protein 2/3 (*hrp2/3*) gene [7, 8] and emergence as well as spread of invasive *Anopheles stephensi* vectors [9, 10]. There are also non-biological threats, such as reduced funding for malaria control, climate change and political will at the global and local/country levels, and these need to be urgently addressed to get progress back on track [11, 12].

In Tanzania, over 93.0% of the population is at risk of malaria because they live in areas where transmission occurs [13]. Due to scaled-up interventions, the country has recently transitioned from very high and stable to areas with varying and/or unstable transmission intensities [14, 15]. As in many malaria-endemic countries in the WHO-Afro region, *Plasmodium falciparum* is the cause of most of the infections in Tanzania, with other species being *Plasmodium ovale* spp. and *Plasmodium malariae* [16–18]. Although *Plasmodium vivax* has been reported in a few studies in Tanzania [16, 18], its public health significance remains unknown. With the current changes in transmission intensities of *P. falciparum*, the dynamics and patterns of non-falciparum species will need to be closely monitored to ensure they do not become new threats to malaria elimination especially in WHO-Afro where they are currently less predominant.

To accelerate progress toward its elimination targets by 2030, Tanzania adopted the WHO’s recommendations and has been implementing effective interventions over the past two decades to control and eventually eliminate malaria. The Tanzanian National Malaria Control Programme (NMCP) has endeavoured to ensure high coverage and use of such interventions, focusing on vector control, case management and preventive therapy. Vector control interventions include the use of insecticide-treated nets (ITNs), indoor residual spraying (IRS) and larval source management (LSM). For effective case management, NMCP uses RDTs for parasitological confirmation of malaria parasites and artemisinin-based combination therapy (ACT) to effectively treat patients with confirmed infections. To prevent malaria in pregnancy, NMCP deployed and uses intermittent preventive treatment (IPTp) of malaria during pregnancy with sulfadoxine-pyrimethamine (SP) and strives to increase the proportion of women receiving two or more doses of IPTp-SP to reach the WHO target of 80.0% [19]. These interventions yielded positive results, with a declining trend of malaria burden which was associated with a decline of prevalence from 18.0% in 2008 to 8.0% in 2022 [20].

Based on recent reports, malaria remains a major public health problem in Tanzania, and the country still experiences persistent transmission in some regions [2], but the drivers of these patterns are not clearly known. Malaria transmission in Tanzania has become heterogeneous, with a high burden in the north-western, southern and western regions, while the central corridor, north-eastern and south-western parts of the country have low to very low transmission intensities [21, 22]. The recent epidemiological transition and trends of malaria have raised critical questions that need to be addressed to allow the country to progress to its elimination targets by 2030. In addition, Tanzania faces emerging and endemic challenges, such as low coverage of existing interventions, emergence and spread of drug and insecticide resistance and weakness of the public health system [23]. Most of these challenges are particularly context-specific and require innovative and multifaceted approaches to ensure that high impact is attained and sustained. The interventions should also target and aim to eliminate the remaining pockets in low transmission areas, respond to biological threats, target non-falciparum species, reduce the disease burden in high transmission areas and address populations at higher risk [24]. Thus, the NMCP needs to strategically and innovatively continue to implement current and new WHO-recommended interventions, including upgrading and making malaria surveillance a core intervention to facilitate ongoing elimination efforts in all areas based on the local burden and level of transmission intensities [25].

Tanzania faces biological and other threats that may compromise its progress and prospects to eliminate malaria by 2030 [26]. Of the biological threats, Tanzania has reported a high prevalence of mosquitoes with resistance to multiple insecticides [4, 27], and it is at high risk of the invasive *An, stephensi* vector, which has been reported in Kenya and the Horn of Africa [28]. In addition, artemisinin partial resistance (ART-R) has been reported in some parts of Kagera region near the border with Rwandan and Uganda, with an average prevalence of Kelch 13 R561H mutation of 7.7% among symptomatic patients and a high prevalence in Karagwe (22.8%) and Kyerwa district (14.4%) [29, 30]. These parasites can potentially spread to other regions if not curtailed, suggesting that a rapid response strategy is urgently needed [29]. Although recent studies have reported a low prevalence of parasites with *hrp2/3 gene* deletions associated with the failure of HRP2-based RDTs [8], these threats are still imminent, and the country needs to increase its vigilance through a strong surveillance system. It is also critical to urgently adopt and implement the WHO response strategy for dealing with ART-R [31] to prevent the spread of resistant parasites from Kagera to other regions.

According to the WHO strategy for responding to ART-R [31], Tanzania needs to develop and implement a response plan customized to the local context and coordinated with neighbouring countries in the Great Lakes region of Africa (Burundi, Democratic Republic of Congo—DRC, Rwanda and Uganda). Since Kagera is one of the regions with a high burden of malaria [32], it is also important to deal with other critical issues, including asymptomatic malaria infections. Due to high transmission intensities in Kagera, asymptomatic infections are potentially another threat that may play a critical role in sustaining and perpetuating transmission of the disease in the region. Studies have shown that individuals with parasites but no apparent symptoms, tend to harbour parasites for a long time without seeking medical care and become silent reservoirs, capable of transmitting the infections to others through mosquito bites [33]. The silent nature of asymptomatic infections which are normally associated with low parasite densities, makes these infections challenging to diagnose and treat promptly, allowing the parasites to persist in the human population [34]. The continuous circulation of parasites among asymptomatic individuals not only poses a threat to their health by potentially progressing into symptomatic cases but also increases the overall transmission potential [35, 36]. These asymptomatic carriers contribute significantly to the persistence of malaria and complicate the efforts to control and eliminate malaria.

In areas like Kagera, where there is a high burden of malaria, emerging reports of ART-R and potentially high prevalence of asymptomatic infections, it is not known yet how resistant parasites will evolve over space and time. Thus, targeting and addressing the high burden of malaria including asymptomatic infections through comprehensive surveillance and intervention strategies are essential in the response to ART-R. This will involve and depend on the ability to detect all malaria infections at health facilities and in the community, and the use of effective interventions to break the cycle of transmission and achieve sustainable malaria control goals [37]. This study assessed the prevalence and predictors/risk of malaria infections among asymptomatic individuals living in an area with a high prevalence of malaria infections [38] and a high prevalence of parasites with ART-R in Kyerwa District, north-western Tanzania as recently reported [29]. The study fills a critical gap by providing data on the magnitude and prevalence of infections in asymptomatic individuals, which serves a potential role in understanding transmission dynamics and risk of infection, including the potential spread of resistant parasites. This study also generated important data that will support the understanding of the burden and risk of asymptomatic malaria infections as well as the epidemiology of parasites with ART-R. The findings will potentially help the government and other stakeholders in planning and implementing strategies for malaria prevention and control as well as responding to the threat of ART-R in Kagera region.

## Methods

### Study design and sites

**Fig. 1:**
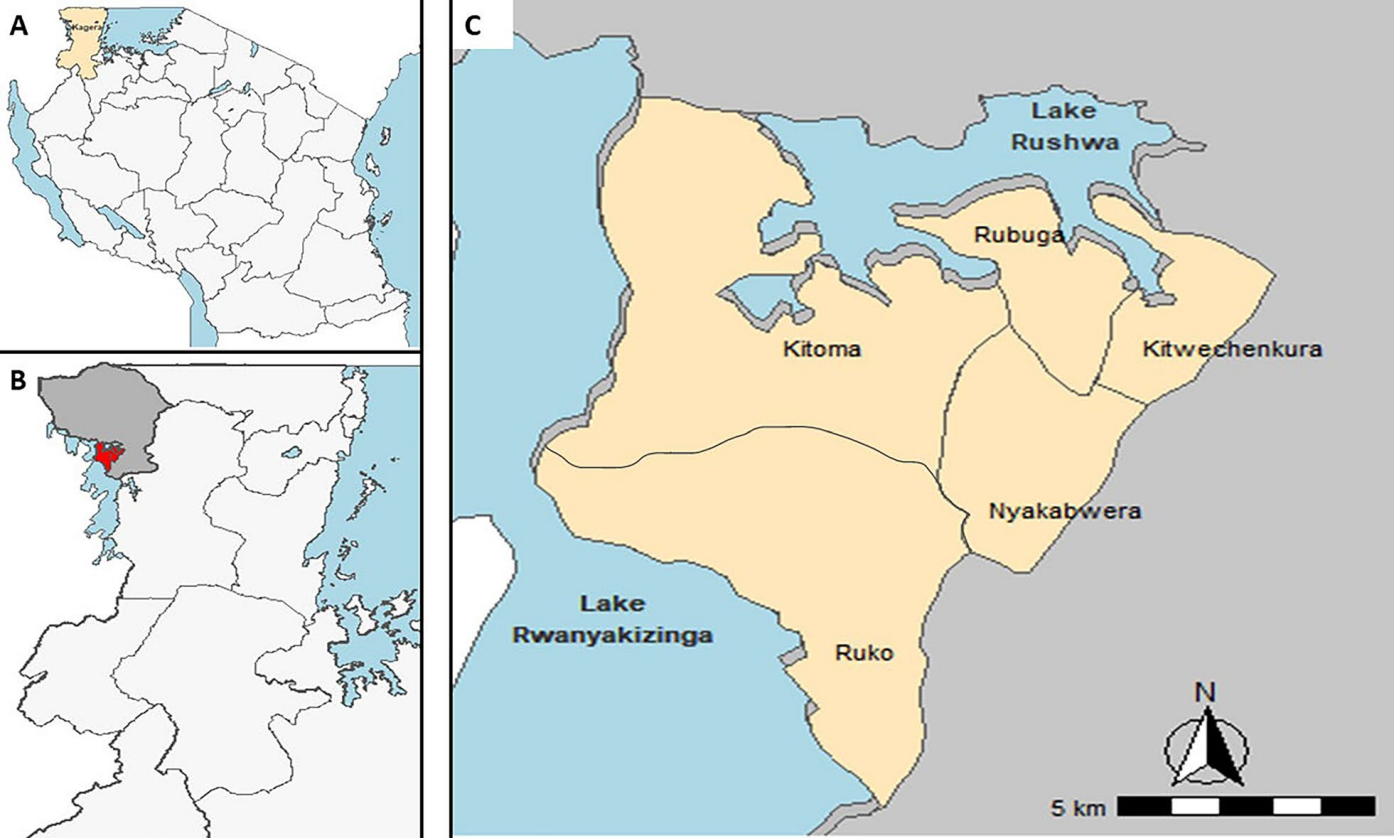
Maps showing the 26 regions of Tanzania including Kagera (gold) (**A**), study villages (red) in Kyerwa district in grey (**B**) and the five study villages (**C**)

This was a community-based cross-sectional survey (CSS) that was conducted during the peak of malaria transmission season in July and August 2023. The timing of this CSS was based on experience from previous studies conducted in other parts of Tanzania and elsewhere which showed that the peak of malaria transmission occurs after the rainy season and provides the best estimate of malaria burden in the target area [39, 40]. The survey was done as part of the main project on molecular surveillance of malaria in Tanzania (MSMT), which has been running in 13 regions of mainland Tanzania since 2021 [8, 29, 41] and was recently extended to cover all 26 regions in 2023 (Ishengoma D—unpublished data). The study covered five villages (Kitoma, Kitwechenkura, Nyakabwera, Rubuga and Ruko), which are part of the longitudinal component of the MSMT project that was initiated in 2022 to monitor the trends and patterns of malaria in areas with special features such as ART-R and *hrp2/3* gene deletions as described elsewhere [8, 29, 30]. The study villages are located in Kyerwa district, which is one of the eight districts of Kagera region (Fig. 1). Kyerwa district covers an area of 3,086 km^2^ and borders Uganda to the North, Rwanda to the West and Karagwe district to the South. The study villages are located near the Kagera River basin, which forms the boundary between Kyerwa and Rwanda (West) and Uganda (North). According to the 2022 national census, Kyerwa district had a population of 412,910 people, including 211,685 females and 201,225 males, with an average household size of 4.3 and a sex ratio of 95 males per 100 females [42].

### Study population and recruitment of participants

This CSS included individuals aged ≥ 6 months living in the 5 villages that are part of the longitudinal component of the MSMT project and provided consent to participate in the study. The survey aimed and targeted to recruit approximately 30.0% of all individuals from each village who were registered during a census survey that was conducted in February 2023. In this survey, all community members meeting the inclusion criteria were considered to be asymptomatic for malaria because they were not at the health facility located in Kitwechenkura seeking medical care for malaria or any other illnesses. They were all invited to participate in the study, but those who wanted to participate were required to meet the inclusion criteria for the survey, including residence in the study villages, age from 6 months and above, and providing informed consent. Individuals living in other villages or those who declined to give informed consent were excluded from the CSS. The information about the CSS was passed to all household members by a community organizer who broadcasted using a loudspeaker and walked through all parts of the village. Each of the study villages had 3–5 hamlets, and members from 1 to 3 hamlets were invited to meet the study team on specific days during the survey. The study team was stationed at a central location that was set up as a CSS post in each village. Any member who missed out was allowed to visit the survey post on a different day in the same or the next village. Thus, participants were recruited conveniently based on their willingness to take part in the study by visiting the recruitment post and providing consent to take part in the CSS.

### Data collection procedures

#### Household census

**Fig. 2:**
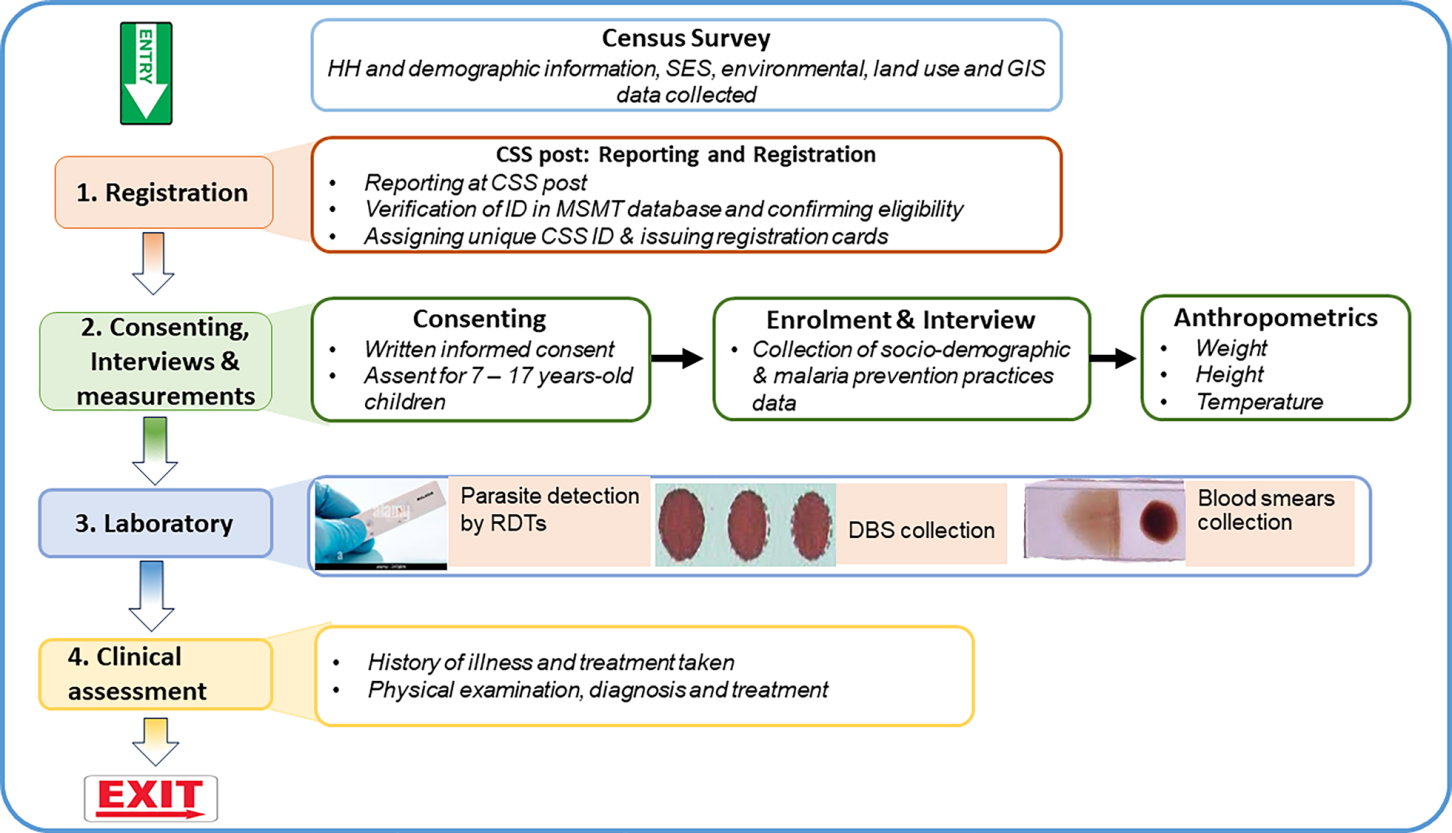
Schematic drawing showing the flow of study participants during the cross-sectional community survey [*CSS* Cross-sectional survey, *DBS* dried blood spot on filter paper, *HH* household, *ID* Identification numbers, *GIS* Global positioning system, *MSMT* = molecular surveillance of malaria in Tanzania project, *RDT* rapid diagnostic test and *SES* socio-economic status

Prior to the CSS, a household census survey was conducted in February 2023 in the study villages. The data collected included demographic, anthropometric, clinical and parasitological data, as shown in Fig. 2. Initially, basic demographic data were collected during the census survey that covered all households and individuals in each family as previously described [43]. Researchers/trained assistants visited the household during the census and every member of the community was enumerated and given a unique identification number (ID). Demographic information, education status and occupation of all members of the household were collected, together with information on malaria control including the use of bed nets or other methods to control mosquitoes, such as repellents or burning herbs. Global Positioning System (GPS) coordinates of each house were recorded and observations were made to record information about the type of house, construction materials used, and presence of windows and eaves. Any other physical features of the house and around it which could support the breeding of mosquitoes (such as vegetation cover and the presence of bushes around the house), or access to humans by mosquitoes when feeding was also recorded. Additional information including land use for agriculture and/or keeping livestock, and vegetation cover was collected during the census survey from each household. While at the household, details of ownership and use of bed nets were assessed and verified by recording the number of nets and confirming where nets were hung at night. The nets were physically inspected to determine their intactness or extent of damage which involved reporting numbers and size of holes on the nets using the methods reported earlier [44]. Ownership of different assets was also recorded for determining the family’s socioeconomic status (SES) as previously reported [45]. A database of all community members was set up at the National Institute for Medical Research (NIMR) in Dar es Salaam.

#### Recruitment and evaluation of participants

During the CSS, each participant was identified using their IDs, which are linked to the main MSMT database containing all community members. After confirming the identity of the prospective participants, they were provided with study identification numbers that were generated specifically for the CSS. Thereafter, participants were interviewed to obtain demographic information on top of those collected during the census, a quality-control step that is built into the CSS to help verify census data. Each study participant was asked to report malaria prevention practices through a series of questions, including ownership and use of bed nets (ITN) the night before the survey. They were also asked to report how frequently other family members used bed nets, their habit of checking and repairing bed nets, and the use of other methods of malaria prevention such as mosquito spray/repellent within their household. Thereafter, they were sent to a designated area for the collection of anthropometric measurements (weight, height and body temperature) and then to the laboratory to test for malaria and collection of blood samples. For the detection of malaria parasites, participants were screened using RDTs, which included Abbott Bioline Malaria Ag Pf/Pan (Abbott Diagnostics Korea Inc., Korea) and Smart Malaria Pf/Pan Ag Rapid Test (Zhejiang Orient Gene Biotech Co. Ltd, China). All recruited participants donated dried blood samples on filter paper (DBS) for laboratory analyses, which will be reported elsewhere. Blood slides for the detection of malaria parasites by microscopy were also collected for reading in the laboratory, but the results are not included in this paper. The final step involved clinical assessment and collection of data on the history of any illness and diagnosis of any presenting illnesses. Additional data including the use of anti-malarials and any other medications which might have been used together with anti-malarials or for the management of other illnesses was collected. Any participant who tested positive by RDT was treated with artemether-lumefantrine (AL) alone and/or other drugs if they had other illnesses with or without malaria (Fig. 2).

### Malaria control interventions in the study villages

This is the first CSS to be conducted in these villages based on the methods that have been used previously in selected communities of Tanga region since 1992, as described elsewhere [46, 47]. Thus, an assessment of malaria control interventions was done to report the use of key interventions recommended by NMCP to account for the impact of the interventions on the burden of malaria in these villages. The five villages are served by the dispensary located in Kitwechenkura village where malaria case management services are provided, based on the national guidelines [48]. The services include early detection of malaria parasites by RDTs and effective treatment with AL. Pregnant women are tested for malaria at the first antenatal care visit and those with positive RDTs are treated with AL. Information obtained from the district malaria focal person (DMIFP) and through a visit to the dispensary showed that all commodities such as RDTs, anti-malarials and other related medicines were available and stock out of these essential commodities was uncommon. It was also reported that all medical services for malaria are provided free of charge in the public health facilities for under-fives, pregnant women and elderly. Other members of the community have to pay cash or use the community health fund (CHF) for medical care at the public dispensary and other health facilities in case of referral. However, the CHF has been reported to have major limitations to users such as the inability to afford by many people, poor services to subscribers and others which have been described elsewhere [49]. In the CSS, all participants mainly asymptomatic (and a few symptomatic) were tested with RDTs, and those with positive results were treated with AL for uncomplicated malaria. Participants with malaria and other illnesses and based on clinical indication were treated with AL and other drugs according to the national guidelines [50]. The services provided during the CSS were free to all participants as compensation and incentive for taking part in the survey.

Through interviews with the DMIFP and during the census survey, information about sources of ITNs and the use of other vector control interventions, such as IRS and LSM, was collected. It was reported that Kyerwa receives bed nets from the NMCP through different campaigns, such as mass distribution, antenatal care (ANC) clinics and school net programmes [51, 52], together with private distributors. According to the NMCP reports of 2022, bed-net ownership in Kyerwa was 89.0% and use was > 80.0% (NMCP Unpublished data). During the CSS, the assessment of vector control methods involved asking each participant to report on ownership of bed nets and their use as well as any information about the use of other methods to kill or repel mosquitoes. The study villages and other parts of Kyerwa district and the entire region of Kagera were covered by the IRS programme funded by the US President’s Malaria Initiative (PMI) from 2008 to 2013 [53, 54]. The villages were also involved in bio-larviciding operations using bio-larvicides produced in Tanzania between 2016 and 2020, but these activities could not be sustained because of a lack of funding. Additional information about surveys of malaria vectors was explored from the DMIFP and other sources, indicating that the villages took part in entomological surveillance in the past.

### Data management and analysis

The data were collected during census and CSS with structured questionnaires created using Open Data Kit (ODK) software, installed and run on tablets. The data were directly transmitted to a central server located at NIMR in Dar es Salaam. The data were downloaded into Excel, cleaned and then transferred to STATA version 13 (STATA Corp Inc., TX, USA) for further cleaning and analysis. Descriptive statistics including frequency, mean, standard deviation (SD) and median (with interquartile (IQR)) were used to summarize data. Chi-square test was used to compare categorical variables while continuous variables were compared using Student’s t-test and Mann–Whitney test. Binary and multivariate logistic regression was used to assess the association between malaria prevalence and different independent variables including demographics and clinical covariates such as sex, age group, use of bed nets, history of fever and fever at presentation (axillary temperature  ≥ 37.5 °C).

Furthermore, the association between the prevalence of malaria with house characteristics such as socio-economic status (SES), family size, type of eaves, type of windows, type of floor and walls, vegetation cover and the presence of bushes around the houses was investigated. The SES for the wealth index was computed using principal component analysis (PCA) as described elsewhere [45]. In the PCA, the scores from the first component of PCA were extracted and then categorised into three wealth quantiles (low, medium and high). The households falling in the low quantile represented poor households, while those in the high quantile represented households with better SES. Variables that were statistically significant (p-value < 0.25) during univariate logistic regression were retained and included in multivariable logistic regression. During the analysis, two separate models were used: the first model included demographic and clinical variables, while the second model included household and environmental characteristics and cluster standard errors to account for clustering at the village level. Multicollinearity was assessed between pairs of independent variables using variance inflation factor (VIF), and those variables with VIF > 5 were excluded from the multivariate model. A p-value of ≤ 0.05 was considered to be significant.

## Results

### Baseline characteristics of study participants

A total of 4454 individuals constituting 29.9% (n = 15031) of all community members participated in the CSS from the five villages and most of them were females (59.3%). The median age of participants was 14 years (IQR: 7–36 years), with significant differences in the age of participants among the study villages (p < 0.01). The majority of participants were students/children (49.5%) and 48.8% were peasants or fishermen. Of the adults (aged  ≥ 15 years), 42.8% had completed primary education, while 30.5% did not have formal education. Of all participants (n = 4454), 29.4% (n = 1310) reported a history of fever in the past 48 h before the survey, but only 3.1% (136/4454) had a fever at presentation (with axillary temperature ≥ 37.5 °C) (Table 1). The majority of the participants (79.7%) were living in households with ≥ 4 members and low/medium SES (68.0%), and their houses were poorly constructed with open windows and floors made of sand (Table 2). A detailed comparison of the sampled participants and the entire population in the five villages is presented in Supplementary Table 2.

**Table 1:** Baseline characteristics of study participants from the five villages of Kyerwa district of Kagera region

Variable	Total	Kitoma	Kitwechenkura	Nyakabwera	Rubuga	Ruko	p-value
Total population	15031	3257	2128	4371	3129	2146	
Enrolled, n (%)	4454 (29.6)	709 (21.8)	769 (36.1)	1243 (28.4)	974 (31.1)	759 (35.4)	
Sex, n (%)							
Male	1812 (40.7)	317 (44.7)	310 (40.3)	494 (39.7)	390 (40)	301 (39.7)	0.287
Female	2642(59.3)	392 (55.3)	459 (59.7)	749 (60.3)	584 (60)	458 (60.3)	
Age in years							
Median (IQR)	14 (7–36)	16 (8–38)	15 (7–37)	14 (6–35)	12 (5–33)	14 (7–36)	< 0.001
Age group, n (%)							
< 5 years	835 (18.7)	154(21.7)	131 (17.0)	205 (16.5)	180(18.5)	165(21.7)	< 0.001
5≤10 years	772 (17.3)	112 (15.8)	105 (13.7)	214(17.2)	193(19.8)	148(19.5)	
10≤15 years	700 (15.7)	93(13.1)	125 (16.3)	198 (15.9)	158(16.2)	126(16.6)	
≥ 15 years	2147 (48.2)	350(49.4)	408(53.0)	626(50.4)	443 (45.5)	320 (42.2)	
Education level^a^, n (%)	1886	309	383	564	364	266	
None	576 (30.5)	85 (27.5)	66 (17.2)	216 (38.3)	108 (29.7)	101 (38.0)	< 0.001
Incomplete primary	331 (17.6)	52 (16.8)	48 (12.5)	118 (20.9)	65 (17.9)	48 (18.0)	
Primary education	808 (42.8)	150 (48.5)	207 (54.1)	197 (34.9)	152 (41.8)	102 (38.3)	
Incomplete secondary	98 (5.2)	15 (4.9)	37 (9.7)	17 (3.0)	28 (7.7)	1 (0.4)	
Completed secondary	66 (3.5)	5 (1.6)	22 (5.7)	15 (2.7)	11 (3.0)	13 (4.9)	
College (certificate/diploma)	5 (0.3)	2 (0.7)	2 (0.5)	0 (0.0)	0 (0.0)	1 (0.4)	
University education	2 (0.1)	0 (0.0)	1 (0.3)	1 (0.2)	0 (0.0)	0 (0.0)	
Occupation							
Peasant/fisherman	2174 (48.8)	364 (51.3)	366 (47.6)	597 (48)	480 (49.3)	367 (48.4)	0.554
Business^b^	40 (0.9)	7 (1.0)	7 (0.9)	9 (0.7)	13 (1.3)	4 (0.5)	
Employed	36 (0.8)	4 (0.6)	8 (1.0)	11 (0.9)	4 (0.4)	9 (1.2)	
Student/child	2204 (49.5)	334 (47.1)	388 (50.5)	626 (50.4)	477 (49)	379 (49.9)	
Height, mean (SD)	134 (42)	131 (32)	140 (49)	137 (55)	132 (31)	130 (31)	< 0.001
Weight, median (IQR)	36 (18–52)	37 (17–53)	42 (21–54)	39 (20–52)	33 (18–51)	29 (16–50)	< 0.001
History of fever, n (%)	1310 (29.4)	295 (41.6)	162 (21.1)	133 (10.7)	432 (44.4)	288 (37.9)	< 0.001
Temp °C, mean (SD)	36.1 (0.8)	36.2 (0.9)	36.2 (0.7)	36.0 (0.8)	36.1 (0.8)	36.1 (0.7)	< 0.001
Fever (temp ≥ 37.5 °C)	1368 (3.1)	30 (4.2)	21 (2.7)	23 (1.9)	41 (4.2)	21 (2.8)	0.006

*IQR* interquartile range, *n* number of participants, *SD* standard deviation, *temp* axillary temperature,  °*C* degree Celsius, and *%* percentage

^a^Study participants with ≥ 15 years were considered to be old enough and expected to have completed at least primary education, and were therefore assessed for their education level; ^b^Business = mainly small and petty business done in the community

**Table 2:** Characteristics of houses and related environmental factors in the study villages

Variables	Total	Kitoma	Kitwechenkura	Nyakabwera	Rubuga	Ruko	p-value
^c^Individuals assessed in CSS^a^	4128	670	733	1158	875	692	
^c^Houses assessed during census^b^	995	163	189	271	208	164	
Family size							
< 4 people	838 (20.3)	146 (21.8)	157 (21.4)	236 (20.4)	159 (18.2)	138 (19.9)	0.453
≥ 4 people	3290 (79.7)	524 (78.2)	576 (78.6)	922 (79.6)	716 (81.8)	554 (80.1)	
Type of eaves							
Open	1274 (30.9)	236 (35.2)	228 (31.1)	328 (28.3)	181 (20.7)	301 (43.5)	< 0.001
Closed	2854 (69.1)	434 (64.8)	505 (68.9)	830 (71.7)	694 (79.3)	391 (56.5)	
Type of windows							
Closed	728(17.6)	111 (16.6)	166 (22.6)	179 (15.5)	181 (20.7)	91 (13.2)	< 0.001
Open	3010 (72.9)	495 (73.9)	536 (73.1)	895 (77.2)	618 (70.6)	466 (67.3)	
Partially open	239 (5.8)	38 (5.7)	26 (3.5)	60 (5.2)	59 (6.7)	56 (8.1)	
No window	151 (3.7)	26 (3.9)	5 (0.7)	24 (2.1)	17 (1.9)	79 (11.4)	
Type of floor							
Soil/sand	3369 (81.6)	516(77.0)	566 (77.2)	1,046 (90.3)	661 (75.5)	580 (83.8)	< 0.001
Cement	759(18.4)	154 (30.0)	167 (22.8)	112 (9.7)	214 (24.5)	112 (16.2)	
Socio-economic status (SES) of HH							
Low	1479 (35.8)	266 (39.7)	199 (27.1)	415(35.8)	305(34.9)	294 (42.5)	< 0.001
Medium	1330 (32.2)	197(29.4)	268 (36.5)	334 (28.9)	320 (36.6)	211 (30.5)	
High	1319 (32.0)	207 (30.9)	266 (36.4)	409 (35.3)	250(28.5)	187 (27.0)	
Vegetation around the house							
No vegetation	356(8.6)	46 (6.9)	58 (7.9)	137 (11.8)	68 (7.8)	48 (6.9)	< 0.001
Short vegetation	1586 (38.4)	307 (45.8)	192 (26.2)	510 (44)	226 (25.8)	349 (50.4)	
Tall vegetation	2186 (53)	317 (47.3)	483 (65.9)	511 (44.1)	581 (66.4)	295 (42.6)	
Presence of bushes around the house							
Yes	2026 (49.1)	351 (52.4)	308 (42.0)	514 (44.4)	490 (56.0)	363 (52.5)	
No	2102 (50.9)	319 (47.6)	425 (58.0)	644 (55.6)	385 (44.0)	329 (47.5)	< 0.001

^a^Individuals who were recruited in the CSS and had their households assessed during the census. Among all participants, 326/4454 (7.3%) individuals had missing household characteristics

^b^Number of houses where study participants came from, and these houses were assessed during the census survey in February 2023

^c^Number of individuals/houses

*HH* household

### Prevalence of malaria

**Fig. 3:**
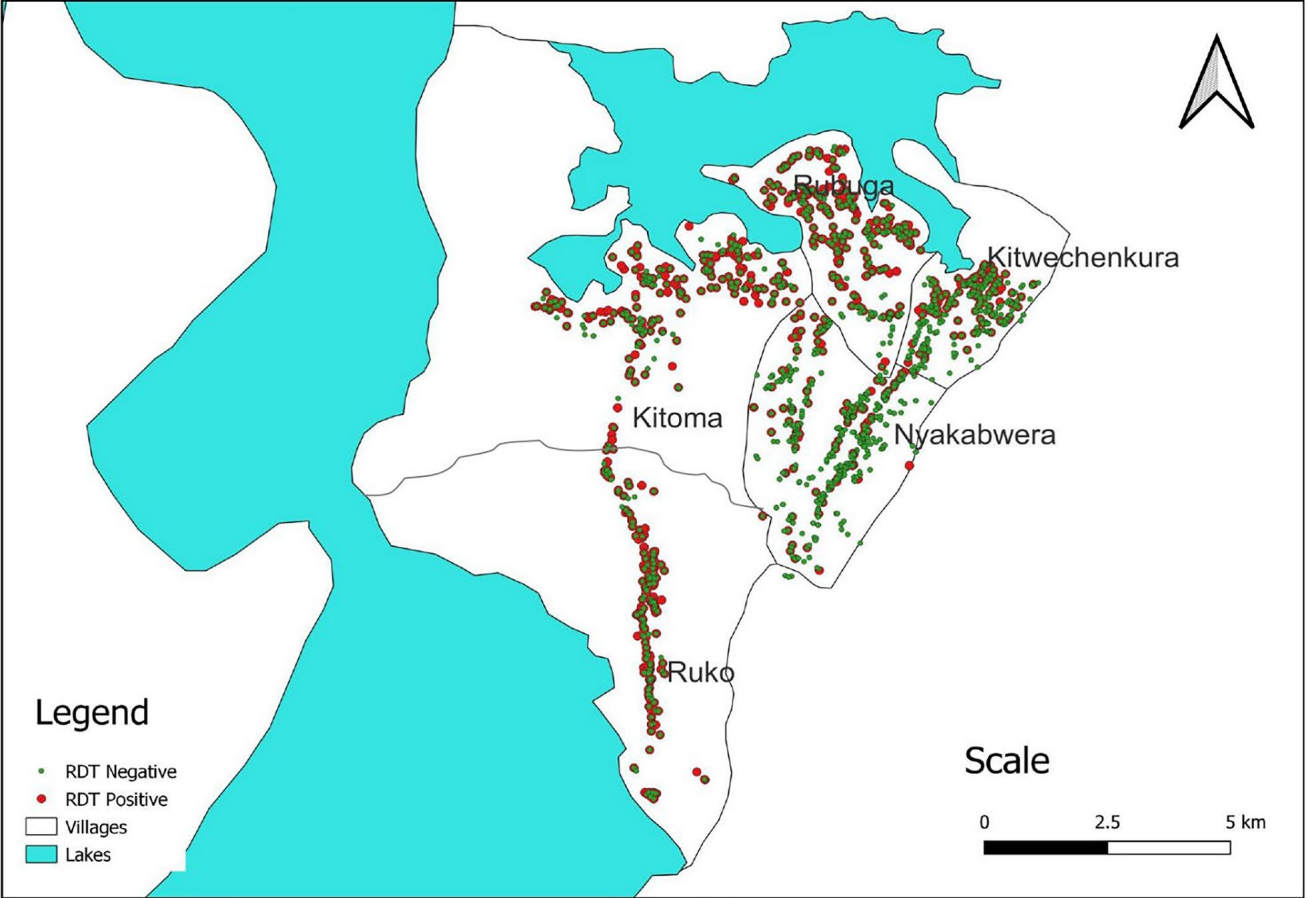
Map of study villages showing the distribution of individuals with malaria parasites detected by rapid diagnostic tests. *RDT* rapid diagnostic test and RDT results are presented by green dot for negative and red for positive test results.

All individuals (n = 4454) were tested with RDTs, and 1979 (44.4%) had positive results. The prevalence of malaria infections in the five villages varied from 14.5% in Nyakabwera to 68.5% in Ruko village and the difference was statistically significant across the villages (p < 0.001). A significantly higher prevalence was detected in males (49.2%) (p < 0.001) in the three villages of Kitoma, Nyakabwera and Rubuga, and among school children in all villages; with the overall prevalence of 61.1% in school children aged 5–≤10 years and 57.4% among those aged 10–≤15 years (Kitoma, Rubuga and Ruko with p < 0.001; Kitwechenkura, p = 0.001 and Nyakabwera, p = 0.037). The lowest prevalence was in adults aged ≥ 15 years (30.4%), and the pattern of age-specific prevalence was similar in all villages (Table 3). The majority of study participants with malaria infections were found in Ruko, Kitoma, Rubuga and Kitwechenkura villages as shown in Fig. 3.

**Table 3:** Prevalence of malaria among individuals of different sex and age groups in the five villages of Kyerwa district

Variable	Kitoma	Kitwechenkura	Nyakabwera	Rubuga	Ruko	Total
Total enrolled (n)	709	769	1,243	974	759	4454
Positive by RDTs, n (%)	440 (62.1)	241 (31.3)	180 (14.5)	598 (61.4)	520 (68.5)	1979 (44.4)
Prevalence by Sex, n (%)						
Male	217/316 (68.7)	99/310 (31.9)	95/494 (19.2)	265/390 (68.0)	214/302 (70.9)	890/1812 (49.2)
Female	223/393 (56.7)	142/459 (30.9)	85/749 (11.4)	333/584 (57.0)	306/457 (67.0)	1089/2642 (41.2)
p-value	0.002	0.770	< 0.001	0.001	0.214	
Prevalence by age groups, n (%)^a^						
< 5 years	121/154 (78.6)	40/131 (30.5)	30/205 (14.6)	130/180 (72.2)	131/165 (79.4)	452/835 (54.1)
5–< 10 years	99/112 (88.4)	47/105 (44.8)	36/214 (16.8)	162/193 (83.9)	128/148 (86.5)	472/772 (61.1)
10–< 15 years	83/93 (89.3)	48/125 (38.4)	39/198 (19.7)	120/158 (76.0)	112/126 (88.9)	402/700 (57.4)
≥ 15 years	137/350 (39.1)	106/408 (26.0)	75/626 (12.0)	186/443(42.0)	149/320(46.6)	653/2147(30.4)
p-value	< 0.001	0.001	0.037	< 0.001	< 0.001	

^a^The prevalence was significantly higher in school children (aged 5–≤10 and 10–≤15 years) than in under-fives and adults in the other four villages (p < 0.001 in Kitoma, p = 0.001 in Kitwechenkura, p < 0.001 in Rubuga and p < 0.001 in Ruko compared to Nyakabwera). RDTs = rapid diagnostic tests, n = number of participants and % = percentage

### Bed net use in the study villages

**Fig. 4:**
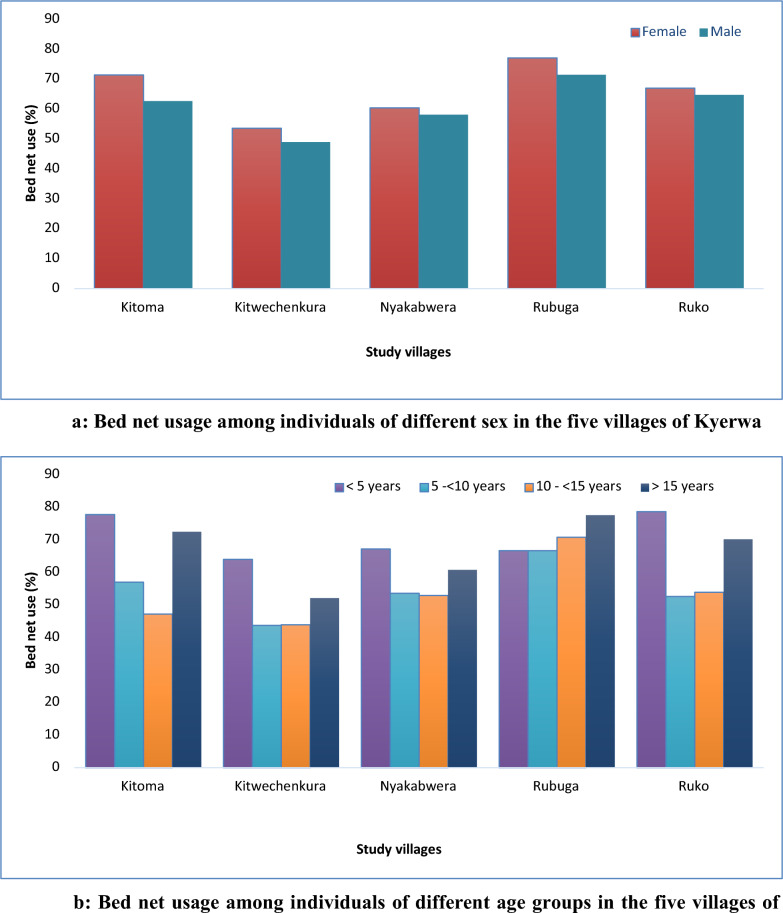
Bed net usage among individuals of different sex in the five villages of Kyerwa. **b** Bed net usage among individuals of different age groups in the five villages of Kyerwa

Table 4 shows the use of bed nets the night before the survey and other methods for malaria prevention among study participants. The overall bed net use (in all villages combined) was 63.9%, with significantly higher usage among females (65.6%) compared to males (61.5%) in all villages (combined p < 0.01). Bed net usage was higher in Rubuga (74.9%) and Kitoma (68.0%), while the lowest was reported in Kitwechenkura (51.8%). There was low bed net usage among school children (56.0% and 54.9% in children aged 5–≤10 and 10–≤15 years, respectively) compared to under-fives and adults (≥ 15 years) with usage of ≥ 73.8% (Table 4). In all five villages, females, under-fives and adults (≥ 15 years) had higher usage of nets, with three villages of Kitoma, Ruko and Rubuga approaching or exceeding 80.0% (Fig. 4a, b). Only a small proportion of participants (2.4%) reported the use of other methods to protect themselves against mosquito bites (Table 4). Such methods included mosquito repellents (n = 67, 1.5%), mosquito coils (n = 21, 0.5%) and burning insecticides (n = 18, 0.4%).

**Table 4:** Use of bed nets among study participants

Variable	Kitoma	Kitwechenkura	Nyakabwera	Rubuga	Ruko	Total
Total enrolled (n)	709	769	1243	974	759	4454
Bed net use, n (%)	482 (68.0)	398 (51.8)	739 (59.4)	729 (74.8)	501 (66.0)	2849 (63.9)
Use of other methods^a^	19 (2.7)	40 (5.2)	15 (1.2)	16 (1.6)	16 (2.1)	106 (2.4)
Bed net use by Sex, n (%)						
Female	280/392(71.4)	246/459 (53.6)	452/749 (60.4)	450/584 (77.1)	306/457 (67.0)	1734/2641 (65.7)
Male	202/317 (63.7)	152/310 (49.0)	287/494 (58.1)	729/390 (71.5)	195/302 (64.6)	1115/1813 (61.5)
P value	0.029	0.214	0.429	0.052	0.564	0.005
Bed net use by age groups, n (%)						
< 5 years	120/154 (77.9)	84/131 (64.1)	138/205 (67.3)	144/180 (80.0)	130/165 (78.8)	616/835 (73.8)
5– < 10 years	64/112 (57.1)	46/105 (43.8)	115/214 (53.7)	129/193 (66.8)	78/148 (52.7)	432/772 (56.0)
10- < 15 years	44/93 (47.3)	55/125 (44.0)	105/198 (53.0)	112/158 (70.9)	68/126 (54.0)	384/700 (54.9)
≥ 15 years	254/350 (72.6)	213/408 (52.2)	381/626 (60.9)	344/443 (77.7)	225/320 (70.3)	1417/2147 (66.0)
P value	< 0.001	0.003	0.007	0.006	< 0.001	< 0.001

Use of other protection methods^a^ (mosquito repellents, mosquito coils and burning insecticides), n = number of participants, % = percentage

### Predictors/Risk factors of malaria infections

After adjusting for demographic characteristics, history of fever, fever at presentation (axillary temperature ≥ 37.5 °C) and bed net use, the results showed that the odds of malaria infections were significantly higher in four villages of Kitoma, Kitwechenkura, Rubuga and Ruko compared to Nyakabwera (p < 0.001) (Table 5). Males and individuals who did not use bed nets the night before the survey had higher odds of malaria infections compared to females (p = 0.003), and those who used nets (p = 0.024), respectively. School children and under-fives had significantly higher odds of malaria infections (p < 0.001) than adults (≥ 15 years). The odds of malaria infection were 18 times higher among participants with a history of fever in the past two days before the survey (p < 0.001) and the odds were significantly higher (2.71) in participants with fever at presentation (p < 0.001) (Table 5). In the model that involved adjustments for households and environmental-related factors (shown in Table 2), individuals from households with four or more members had higher odds of malaria infections (p < 0.001), as did those living in houses with open (p = 0.015) or partially open windows (p = 0.002). Also, participants living in houses with no windows (p < 0.001) and individuals from households with low SES (p < 0.001) had significantly higher odds of malaria infections compared to those living in houses with closed windows or from households with high SES, respectively. Moreover, the presence of long vegetation and bushes around houses was associated with higher odds of malaria infections (Table 6).

**Table 5:** Demographic and clinical factors associated with malaria infections in the five villages of Kyerwa

Variable	Crude OR	95% CI	P value	Adjusted OR	95% CI	P-value
Village						
Nyakabwera	Reference			Reference		
Kitoma	9.66	7.76–12.02	< 0.001	9.16	7.00–11.99	< 0.001
Kitwechenkura	2.70	2.16–3.36	< 0.001	2.49	1.92–3.23	< 0.001
Rubuga	9.39	7.66–11.52	< 0.001	7.33	5.71 9.40	< 0.001
Ruko	12.85	10.31–16.01	< 0.001	12.02	9.24–15.63	< 0.001
Age group						
< 5 years	2.70	2.29–3.18	< 0.001	2.06	1.63–2.60	< 0.001
5≤10 years	3.59	3.03–4.27	< 0.001	3.88	3.07–4.91	< 0.001
10≤ 5 years	3.08	2.58–3.68	< 0.001	4.06	3.22–5.13	< 0.001
≥ 15	Reference			Reference		
Sex						
Female	Reference			Reference		
Male	1.37	1.22–1.55	< 0.001	1.28	1.08–1.51	0.003
Bed net use						
Yes	Reference			Reference		
No	1.03	0.91–1.17	0.621	1.22	1.03–1.46	0.024
History of fever*						
No	Reference			Reference		
Yes	22.38	18.58–26.95	< 0.001	18.09	14.70–22.26	< 0.001
Fever (temp ≥ 37.5 ^0^C)						
No	Reference			Reference		
Yes	5.05	3.31–7.68	< 0.001	2.71	1.53–4.80	0.001

An adjustment was done for age group, sex, bed net use, history of fever and fever at presentation

*CI* confidence interval, *OR* Odds ratio, *temp* temperature, and °C degree Celsius

**Table 6:** Household and environmental characteristics associated with malaria infections in the five villages

Variable	Crude OR	95% CI	p-value	Adjusted OR	95% CI	p-value
Family size						
< 4 people	Reference			Reference		
> = 4 people	1.31	1.19–1.44	< 0.001	1.43	1.31–1.55	< 0.001
Type of eaves						
Closed	Reference			Reference		
Open	1.11	0.78–1.58	0.568			
Type of windows						
Closed	Reference			Reference		
Open	1.24	1.01–1.53	0.044	1.30	1.05–1.61	0.015
Partially open	1.27	1.01–1.60	0.041	1.33	1.11–1.58	0.002
No window	2.05	1.37–3.06	< 0.001	2.08	1.46–2.96	< 0.001
Type of floor						
Cement	Reference			Reference		
Soil/sand	0.72	0.49–1.04	0.079	0.58	0.37–0.93	0.023
SES						
Low	1.18	1.08–1.28	< 0.001	1.42	1.17–1.72	< 0.001
Middle	1.15	0.92–1.44	0.213	1.31	0.98–1.75	0.069
High	Reference			Reference		
Vegetations around the houses						
No vegetation	Reference			Reference		
Short vegetations	1.18	0.85–1.65	0.330	1.12	0.84–1.49	0.446
Tall vegetations	1.40	1.01–1.95	0.048	1.30	1.02–1.65	0.032
Presence of bushes around the houses						
No	Reference			Reference		
Yes	1.26	1.05–1.51	0.013	1.25	1.06–1.48	0.008

*CI* confidence interval, *OR* Odds ratio

An adjustment was done for family size, type of windows, type of floor, SES, vegetation and presence of bushes around the houses.

## Discussion

This CSS was conducted as part of a larger project on MSMT that aims to establish the capacity and implement malaria molecular surveillance (MMS) in Tanzania [55]. The MSMT project has been implemented in 13 regions of Tanzania since 2021, and it has now been extended to cover integrated malaria molecular surveillance (iMMS) in all 26 regions of Mainland Tanzania since January 2023 (Ishengoma et al. Unpublished data). This study generated important baseline data in five villages in an area with a high malaria burden [56] and a high prevalence of parasites with ART-R in Kagera region, north-western Tanzania [29]. The study utilized a platform that has been set up by the MSMT project to support studies focused on iMMS in Tanzania. It enrolled asymptomatic individuals from the five villages under the longitudinal surveillance component of MSMT and showed that all villages had a very high prevalence of malaria infections by RDTs (44.4%), with high variability despite their proximity. In these villages, the odds of malaria infections were significantly higher in males, school children, individuals with low SES, and those who were not using bed nets or living in poorly constructed houses. In all villages, the prevalence and odds of malaria infections were heterogeneous at the micro-geographic level but with some evidence of clustering near the lakes, despite the high use of bed nets (over 51.0%), which exceeded 66.0% in three villages. The findings reported here will be critical in future studies to characterize and monitor transmissibility, trends and patterns as well as the spread of parasites with ART-R in Kagera region and other parts of Tanzania. The findings will be particularly useful in the control of asymptomatic cases which are normally not targeted by the current case management interventions.

In this study, the majority of participants were females compared to males, and most of them were farmers or fishermen. In addition, the majority (approximately 70.0%) were either illiterate, did not complete or had primary education, and over 26.0% were schoolchildren. These demographic features present a population of individuals with potentially higher odds of malaria infections due to low SES (shown to be associated with high odds of malaria infections in this study). Preliminary analysis of the census data collected in this community indicates that the majority of the people with low SES had similar features, which included females from households with low SES compared to their male counterparts, reporting low education levels, and being peasants (Challe, unpublished data). Studies conducted elsewhere also reported that women were more likely to take part in research studies due to their role as caregivers and perceived high risk of malaria compared to males [57]. In previous studies, it was also shown that low SES was an important risk factor for malaria infections [45, 58–60], mainly due to the strong association between malaria and poverty [61, 62]. Poor people have a higher risk of malaria infections because they cannot afford the cost of malaria prevention and/or treatment and they lack and/or possess a low level of knowledge and skills required to protect themselves against malaria infections [63].

Although this study targeted asymptomatic individuals from the community, about one-third (29.9%) of the participants reported that they had a fever in the past 48 h, and only 3.1% had a fever at presentation (measured axillary temperature ≥ 37.5 °C). This could be due to overreporting by study participants in anticipation of getting medicines for malaria because all medical services were given for free (C. Mandara, Pers. Commun). In previous studies, it was observed that study participants reported having malaria or any other febrile illnesses because they wanted to obtain better services from the study team and medicines that were given for free. It was also shown that participants reported illness because they would prefer to be given medicines to keep for future use or to share with family members who were not present at the time of the study, or in case they fell sick later. When data analysis was performed to tease out the relationship between reported fever history and the results of RDTs, there was a significant association between a history of fever and malaria infection as reported elsewhere [64, 65]. The high association between fever history in this study could also be due to the high prevalence of malaria in the study villages. Thus, fever history was possibly over-reported by participants in anticipation of receiving better services and medicines. Similarly, there was also a strong association between fever at presentation (axillary temperature ≥ 37.5 °C) and malaria infections, which was similar to the findings of other community studies that showed that fever at presentation was a strong predictor of confirmed malaria infections by RDT and/or microscopy [66].

The overall malaria prevalence in this community was very high (44.4%), with a higher prevalence among males, and in three of the five villages, the prevalence exceeded 61.0%. There was very high heterogeneity across the five villages that are located close to each other, suggesting that there are critical factors associated with the micro-geographic pattern of malaria in this community. In these villages, most of the malaria-infected individuals were clustered around the lakes highlighting the potential role of these water bodies in mosquito breeding and disease transmission. Further studies are warranted to test this observed clustering and understand its causal nature. A recent survey of malaria vectors which was conducted as part of the MSMT has collected a large number of mosquitoes which are being analysed to determine the species composition and their infectiousness (Derua Y, unpublished data). Once these findings are available and when compared to the prevalence of malaria infections reported in this study, it will possibly be clear that most malaria infections in these villages are perpetuated and sustained by the lakes which provide stable breeding sites throughout the year.

The findings of high and variations in the prevalence of malaria infections in asymptomatic individuals in these villages are similar to what was reported elsewhere [56], where a high level of heterogeneity at microgeographic levels was reported within the wards from 80 councils of Mainland Tanzania [67]. Although few studies have assessed the prevalence of malaria in asymptomatic individuals of all age groups in Tanzania, the prevalence reported in this study is likely the highest in the country in recent years. Studies conducted in Tanga reported that the prevalence of malaria among under-fives and school children was higher (≥ 68.0%) between 1992 and 1999 but declined to less than 10.0% in 2012 [68]. Following an increase and a rebound of malaria in Tanga, the prevalence increased, but the highest was 31.4% in 2015 [47]. In Rufiji, a decline in prevalence was also reported, and the highest was 90.0% in 1985, while the surveys conducted between 2001 and 2006 reported the highest prevalence of 23.0% [69]. The causes of this high level of prevalence in these villages of Kyerwa district need to be established to guide specific interventions to reduce the burden of malaria, which will potentially reduce the spread of parasites with ART-R currently circulating in this and other areas of Kagera region.

The age-specific prevalence showed a significantly higher prevalence in school children (aged 5–≤15 years) followed by under-fives, while adults (≥ 15 years) had the lowest prevalence (overall and in each of the five villages). A high prevalence of malaria among school children has consistently been reported in studies conducted in Tanga since 2008 [47] and was attributed to delays in the development of immunity due to declining transmission and reduced exposure to infectious mosquito bites. A similar trend has been reported in other parts of Tanzania, particularly through school surveys. Over the past 10 years, a high prevalence of malaria among school children has been reported, with the prevalence reaching 76.4% in some district councils [21]. However, a declining trend of malaria in school children has also been observed in school surveys conducted from 2015 to 2021, with an overall decline from 21.7% in 2015 to 11.3% in 2021 (Chacky et al. Pers. Commun.). In this study, the observed high prevalence of malaria among school children, which was ≥ 75.5% in the three villages (Kitoma, Rubuga and Ruko), needs to be monitored to determine the factors associated with this high burden of malaria in this group. Future studies will also need to focus on the potential lack or low impact of different interventions against malaria in this community, specifically those directed at school children.

Three of the five villages had high bed net use (over 66.0%), two villages had low use (less than 60.0%), and one village had very low use of bed nets (51.8%). In all villages, bed net use was higher among females than males and significantly lower in school children (overall and in each of the five villages). High bed net use among females is possibly due to ongoing campaigns to provide free bed nets to pregnant women at ANC visits in Tanzania [52]. However, low bed net use by school children could account for the high prevalence of malaria in this group, and this is surprising because a school net programme has been running in Tanzania since 2013 [70]. This campaign aims to control and reduce the burden of malaria in these children. Although previous studies reported an increase in bed net ownership among school children [51, 71, 72], it is possible that ownership could be high, but bed nets are not routinely used. Additional studies are needed to tease out the level of bed net ownership and use in this community and their impacts on the malaria burden since school children who were expected to be protected by nets still had low use and the highest prevalence of malaria.

In all villages, under-fives had significantly higher bed-net use, followed by adults. In three of the five villages, the use of bed nets among under-fives was closer to or exceeded 80%, which is the cut-off recommended by the WHO. However, under-fives had a higher prevalence of malaria in all villages, although it was lower than that observed in school children. It was also shown that a high proportion of adults were using bed nets and, together with high bed net use among under-fives, this could be due to free bed nets given to children and pregnant women attending ANC clinics in Tanzania [73]. However, the lack of impact of high bed net use in young children in this community needs to be further explored. Previous studies emphasized the importance of behaviour change in assessing malaria prevalence, specifically in relation to bed net coverage, ownership and use, because ownership may not be directly related to the use of nets. In addition, it has been shown that outdoor biting mosquitoes are the main cause of infections even in areas with high coverage and use of nets, especially when humans spend time outdoors during the early hours of the night [74]. Therefore, it is crucial to investigate the prevalence of outdoor biting mosquitoes and human behaviour that may expose individuals to malaria infection when outdoors or indoors but not when sleeping under bed nets. Besides the influence of outdoor biting behaviour of some vectors on malaria in areas with high bed net usage, recent studies suggest that mosquito behaviour may change in response to bed net use, and factors such as bed net age, the presence of holes, and the effectiveness of insecticides can play crucial roles [75]. While the initial analysis was not focused on outdoor exposure during early evening hours, the authors acknowledge the need for a more comprehensive exploration of these variables.

The findings of this study showed that the odds of malaria infections were higher in all villages compared to Nyakabwera, among males compared to females and in under-fives and school children compared to adults. It was also shown that individuals who used bed nets had lower odds of malaria infections compared to those who were not using nets, with non-users having higher odds by over 22.0%. Additional risk factors for malaria infections included low SES, living in houses whose floors are made of mud and houses with open windows as well as the presence of tall vegetation and bushes around the house. As shown in previous studies, low SES and poor housing or living conditions are strongly associated with a higher risk of malaria infections [45, 58–60]. This suggests that efforts to eliminate malaria need to target groups at high risk and to consider and concurrently implement strategies for poverty reduction.

Although previous studies have shown high heterogeneity in malaria transmission and risk at the macro-geographic level [56, 76], there is a paucity of studies on micro-geographic variations in malaria burden and associated risk factors [56]. This study highlighted some of the factors but more studies are required to further assess the burden of malaria and the risk factors associated with its transmission at different geographic levels. Additional studies will also be required to determine whether such high transmission will support the spread of resistant parasites in this and other areas of the Kagera region and beyond.

This study had some limitations which may be associated with the use of RDTs, with lower sensitivity compared to molecular methods like PCR, especially in detecting low-level parasitaemia. However, it is crucial to recognize the advantages of RDTs since they provide rapid and cost-effective detection of malaria parasites in field settings, rendering them indispensable tools for prompt detection of malaria infections and large-scale surveillance efforts. The use of convenience sampling is another limitation of this study, as it may introduce bias because participants self-selected themselves based on factors such as accessibility or willingness to participate. Participation could have been possibly influenced by the availability of free medications or services, and opportunities to meet project physicians who are not available in the local dispensary. The study population could have potentially been different in case they were randomly selected from the entire population in the community. This approach might have resulted in a non-representative sample that may not accurately reflect the broader population, thereby limiting the generalizability of the findings. However, the results present similar patterns to what have been reported elsewhere in previous studies possibly suggesting that the selection bias was minimal.

## Conclusion

This study showed a high prevalence of malaria infections and high heterogeneity at the micro-geographic level in five villages located next to each other. Groups with higher odds of malaria infections included school children, males, individuals with low SES, living in poorly constructed houses, and non-bed net users. These findings provide important baseline data in an area with a high prevalence of parasites with ART-R and will be utilized in future studies to monitor the trends and potential spread of such parasites, and in designing an ART-R response strategy as well as interventions targeting asymptomatic malaria infections.

### Supplementary Information

Supplementary Material 1.

Supplementary Material 2.

## Data Availability

The data used in this paper are available and can be obtained upon reasonable request from the corresponding author together with institutional approval by NIMR and signing of a data transfer agreement between the donor and recipient.

## Abbreviations

ACT: Artemisinin-based combination therapy
ANC: Antenatal care clinic
aOR: Adjusted odds ratio
CI: Confidence interval
cOR: Crude odds ratio
CSS: Cross-sectional survey
DMFP: District malaria focal person
IDs: Identification numbers
iMMS: Integrated malaria molecular surveillance
IPTp: Intermittent preventive treatment in pregnant women
IRS: Indoor residual spraying
ITN: Insecticide-treated Net
IQR: Interquartile range
LSM: Larval source management
MMS: Malaria molecular surveillance
MRCC: Medical Research Coordinating Committee
MSMT: Molecular surveillance of malaria in Tanzania
RDTs: Rapid diagnostic tests
NIMR: National Institute for Medical Research
NMCP: National Malaria Control Programme
OR: Odds ratio
ODK: Open Data Kit software
PO-RALG: President’s Office, Regional Administration and Local Government
PCA: Principal component analysis
SES: Socio-economic status
SP: Sulfadoxine-pyrimethamine
WHO: World Health Organization
WHO-Afro: WHO Regional Office for Africa
