# Supplementary material for: High prevalence and risk of malaria among asymptomatic individuals from villages with high prevalence of artemisinin partial resistance in Kyerwa district of Kagera region, north-western Tanzania
Source: Malar J. 2024 Jun 26;23:197. doi: 10.1186/s12936-024-05019-5 (PMC11201325; doi:10.1186/s12936-024-05019-5)
Supplement: Supplementary file 1 — Supplementary Material 1. [file 12936_2024_5019_MOESM1_ESM.docx]

**Supplementary Table 1: Selected assets variables which were used to assess the socioeconomic status, categories and their corresponding scores**

| **Variable** | **Categories** | **SES score** |
| --- | --- | --- |
| Availability of radio | 0=No, 1=Yes | 0.3455 |
| Ownership of motorcycles | 0=No, 1=Yes | 0.3383 |
| Number of acres of land cultivated | 0=None, 1= 1-3=1 2=4 or more | 0.3379 |
| Possession of mobile phones | 0=No, 1=Yes | 0.3332 |
| Number of sleeping rooms/house size | Continuous (1-12) | 0.3198 |
| Source of light | 0 = local lamp[koroboi], 1 = kerosene lump/electricity) | 0.2928 |
| Occupation of head of HH- small business | 0=No, 1=Yes | 0.2224 |
| Toilet - wall type | 0 = Thatch/mud walls, 1 = Bricks) | 0.2155 |
| Source of cooking energy | 0 = Firewood, 1 = charcoal/kerosene/electricity) | 0.1904 |
| Number of sheep/goats | 0 = None, 1 = 1-10, 2 = More than 10) | 0.1902 |
| Possession of bicycles | 0=No, 1=Yes | 0.1422 |
| Number of chickens | 0=None, 1=1-20, 2 =21 or more | 0.1405 |
| Occupation of head of HH -formal employment | 0=No, 1=Yes | 0.1347 |
| Occupation of head of HH - livestock keeping | 0=No, 1=Yes | 0. 1231 |
| Number of cattle | 0=None, 1= 1-4, 2= 5 or more | 0.1126 |
| Ownership of a house | 0=No, 1=Yes | 0.0886 |
| Occupation of head of HH-fishery | 0=No, 1=Yes | 0.0804 |
| Source of drinking water | 1=River/Lake, 2=Tap water /closed well | 0.0028 |

HH = household
