# Supplementary material for: High prevalence and risk of malaria among asymptomatic individuals from villages with high prevalence of artemisinin partial resistance in Kyerwa district of Kagera region, north-western Tanzania
Source: Malar J. 2024 Jun 26;23:197. doi: 10.1186/s12936-024-05019-5 (PMC11201325; doi:10.1186/s12936-024-05019-5)
Supplement: Supplementary file 2 — Supplementary Material 2. [file 12936_2024_5019_MOESM2_ESM.docx]

**Supplemental Table 1**: A comparison of the distribution of individuals’ characteristics between the population and the sample of participants who participated in the cross-sectional survey from the five villages of Kyerwa.

| **Variable** | **Population/Sample ratio** | | | | | |
| --- | --- | --- | --- | --- | --- | --- |
|  | **Kitoma (n=3257)** | **Kitwechenkura**  **(n =2128)** | **Nyakabwera**  **(n = 4371)** | **Rubuga**  **(n = 3129)** | **Ruko**  **(n=2146)** | **Total**  **(N=15031)** |
| Sex |  |  |  |  |  |  |
| Male | 1.1 | 1.2 | 1.2 | 1.2 | 1.2 | 1.2 |
| Female | 0.9 | 0.9 | 0.9 | 0.9 | 0.8 | 0.9 |
| Age group |  |  |  |  |  |  |
| <5 years | 0.7 | 0.9 | 1 | 0.9 | 0.8 | 0.9 |
| 5 - <10 years | 0.9 | 1 | 0.9 | 0.8 | 0.8 | 0.9 |
| 10 - <15 years | 1.1 | 0.8 | 1 | 0.9 | 0.9 | 0.9 |
| ≥15 years | 1.1 | 1.1 | 1.1 | 1.2 | 1.2 | 1.1 |
| Education level |  |  |  |  |  |  |
| None | 1.1 | 1.1 | 0.9 | 1 | 1.1 | 1 |
| Incomplete primary | 1 | 0.9 | 0.9 | 0.8 | 0.8 | 0.9 |
| Primary education | 0.8 | 0.9 | 1.1 | 1.2 | 1.2 | 1 |
| Incomplete Secondary | 1.3 | 1 | 2.2 | 1.5 | 0.8 | 1.4 |
| Complete secondary | 3 | 1.5 | 1.7 | 1.9 | 1 | 1.5 |
| College (certificate/diploma) | 1 | 5.7 | 0 | 0 | 1 | 3 |
| University | 0 | 2 | 1 | 0 | 0 | 1 |
| Occupation |  |  |  |  |  |  |
| Farmer | 1 | 1 | 1 | 1.1 | 1.1 | 1.1 |
| Business | 2.2 | 2.5 | 2.5 | 2.1 | 4.8 | 2.6 |
| Student | 1.1 | 0.9 | 1 | 0.9 | 0.8 | 0.9 |
| Child | 0.8 | 1 | 0.9 | 0.9 | 0.9 | 0.9 |

Most of the sampled participants had a ratio of 1:1 with the population. However, disparities were observed for males, individuals with incomplete or completed secondary education, those with a college education, and those with occupations as farmers or business owners. A similar trend of under-sampling for these characteristics was consistently noted across all villages.
